# Doing battle with “the monster:” how high-risk heterosexuals experience and successfully manage HIV stigma as a barrier to HIV testing

**DOI:** 10.1186/s12939-018-0761-9

**Published:** 2018-04-20

**Authors:** Marya Gwadz, Noelle R. Leonard, Sylvie Honig, Robert Freeman, Alexandra Kutnick, Amanda S. Ritchie

**Affiliations:** Center for Drug Use and HIV Research, Rory Meyers College of Nursing, New York University, 433 First Avenue, 6th floor, New York, NY 10010 USA

**Keywords:** Qualitative, Stigma, Structural, Intersectionality, HIV testing, Heterosexual, Health disparities, African American, Black, Hispanic

## Abstract

**Background:**

Annual HIV testing is recommended for populations at-risk for HIV in the United States, including heterosexuals geographically connected to urban high-risk areas (HRA) with elevated rates of HIV prevalence and poverty, who are primarily African American/Black or Hispanic. Yet this subpopulation of “individuals residing in HRA” (IR-HRA) evidence low rates of regular HIV testing. HIV stigma is a recognized primary barrier to testing, in part due to its interaction with other stigmatized social identities. Guided by social-cognitive and intersectionality theories, this qualitative descriptive study explored stigma as a barrier to HIV testing and identified ways IR-HRA manage stigma.

**Methods:**

In 2012-2014, we conducted in-depth qualitative interviews with 31 adult IR-HRA (74% male, 84% African American/Black) with unknown or negative HIV status, purposively sampled from a larger study for maximum variation on HIV testing experiences. Interviews were audio-recorded and professionally transcribed verbatim. Data were analyzed using a systematic content analysis approach that was both theory-driven and inductive.

**Results:**

Stigma was a primary barrier to HIV testing among IR-HRA. In the context of an under-resourced community, HIV stigma was experienced as emerging from, and being perpetuated by, health care organizations and educational institutions, as well as community members. Participants noted it was “better not to know” one’s HIV status, to avoid experiencing HIV-related stigma, which could interact with other stigmatized social identities and threaten vital social relationships, life chances, and resources. Yet most had tested for HIV previously. Factors facilitating testing included health education to boost knowledge of effective treatments for HIV; understanding HIV does not necessitate ending social relationships; and tapping into altruism.

**Conclusions:**

In the context of economic and social inequality, HIV stigma operates on multiple, intersecting layers. IR-HRA struggle with an aversion to HIV testing, because adopting another stigmatized status is dangerous. They also find ways to manage stigma to engage in testing, even if not at recommended levels. Findings highlight strategies to reduce HIV stigma at the levels of communities, institutions, and individuals to improve rates of annual HIV testing necessary to eliminate HIV transmission and reduce HIV-related racial and ethnic health disparities among IR-HRA.

## Background

Testing for HIV infection is a critical aspect of the strategy to eliminate HIV transmission in the United States [1–3]. Since 2006, the Centers for Disease Control and Prevention (CDC) has recommended at least annual HIV testing for populations considered at high-risk for HIV [4]. Indeed, regular HIV testing is needed to support individuals in their HIV risk-reduction and HIV prevention efforts, and also for early detection of HIV status. Early detection of HIV, in turn, fosters timely initiation of HIV antiretroviral therapy, thereby potentially increasing the effectiveness of antiretroviral therapy, and even extending life expectancy, as well as reducing the chances of forward transmission of HIV to others [5]. Early detection of HIV infection, therefore, has benefits at both individual and community levels. Although the time between HIV infection and first diagnosis has been decreasing in recent years [6], late diagnosis of HIV is still unacceptably common [7]. Not surprisingly, those with the greatest barriers to HIV testing are those most likely to be diagnosed with HIV late in the course of their disease [8]. Thus, increasing rates of regular, annual HIV testing is a public health priority [9].

The present study focuses on a high-risk heterosexuals, a population vulnerable to HIV infection but with relatively low rates of HIV testing compared to other major risk groups such as men who have sex with men. Consistent with the CDC’s National HIV Behavioral Surveillance system, we define high-risk heterosexuals as those socially connected to urban geographical areas with elevated rates of both socioeconomic disadvantage and HIV prevalence, called “high-risk areas” (HRAs). In fact, heterosexual sex is the second most common route of HIV transmission in the United States after male-to-male sexual contact, accounting for an estimated 24% of newly reported infections annually, and is by far the main route of HIV transmission among women. Nationally, fewer than half of all heterosexuals have been tested for HIV at least once in their lives (44.2%) compared to 57.3% among the population of men who have sex with men [10]. In our own research on high-risk heterosexuals in an urban HRA, we found that almost all (93%) had tested for HIV at least once, but only half reported past-year HIV testing (50%) [11]. Further, rates of regular, annual HIV testing, as recommended by the CDC, were low (37%; [11]). Nationally, HIV prevalence is higher among high-risk heterosexuals, who are predominantly African American/Black and Hispanic, than among the underlying general heterosexual population (2.3% vs. 0.6%) [10]. Yet overall heterosexuals are under-studied in comparison to the other major risk groups for HIV such as men who have sex with men and persons who inject drugs [12, 13] .

Since the beginning of the HIV pandemic, HIV-related stigma has been identified as a major deterrent to a range HIV-related protective behaviors including HIV testing, disclosure of HIV status, linkage to HIV care, uptake of HIV antiretroviral therapy, and medication adherence, particularly among vulnerable populations at the greatest risk for HIV infection [14, 15]. HIV stigma often reinforces existing social inequities based on sex, gender, race, ethnicity, socioeconomic status, and sexual minority status [15]. At the individual and interpersonal levels, individuals at risk for HIV, or those encouraged to be tested for HIV, may avoid getting tested, fearing that HIV-related stigma could lead to social isolation, rejection by family members and friends [16–18], discrimination with respect to employment, healthcare, and housing [19–22], and/or victimization [23, 24]. Stigma associated with HIV may be layered upon inequities that operate at the structural, institutional, and cultural levels [25–27]. Structural stigma refers to societal-level conditions, norms, and institutional practices that produce and reinforce the discrimination and exclusion of marginalized groups [28]. These inequities may be woven into the policies and practices of institutional systems including healthcare, employment, housing, education, criminal justice, and infrastructure [29]. Some examples of structural stigma faced by people at risk for HIV include lack of conveniently located HIV testing facilities, lack of high-quality HIV testing experiences, impersonal contact with health care facilities, and physically segregated or largely hidden HIV testing locations in large governmental building complexes [30]. These layered stigmas operate in a multidimensional, or intersectional, manner where structural inequalities are linked to marginalized social positions and identities of race/ethnicity, socioeconomic status, sex, gender, and sexual minority status, which are experienced at the individual level [31, 32]. Thus for heterosexual individuals who already experience the negative effects of race/ethnicity and low socio-economic status, the association with HIV and other stigmatized social categories may threaten to add further to their social vulnerability. This, in turn, may be closely linked to avoidance or fear of HIV testing, especially for those residing in geographical areas with disproportionately high rates of HIV infection [31, 32].

Yet while a substantial literature has used an intersectional framework to examine how structural and social contexts operate as barriers to HIV testing among men who have sex with men from racial/ethnic minority backgrounds [33–38], few studies have examined how these intersecting stigmas shape the experience of HIV testing among heterosexuals at high-risk for HIV [39]. The present study now extends past research on HIV stigma to the population of high-risk heterosexuals, the vast majority of whom are African American/Black and Hispanic and from low socio-economic status backgrounds [39]. In keeping with an intersectional perspective, and because a wide range of socio-demographics characteristics can be used to describe the population, we refer to them as “individuals residing in a HRA” (IR-HRA) in the present study.

Despite the potential of HIV stigma, and intersectional stigma, to impede regular, annual HIV testing among IR-HRA, a substantial proportion has, in fact, received HIV testing, as noted above. Further, in some urban areas at least, a substantial minority test annually [11]. Consistent with these indications of positive health behavior, Earnshaw and colleagues (2013) highlight the importance of identifying modifiable strengths-based moderators of the association between societal stigma and health disparities, and propose a resilience agenda [15].These strength-based modifiable factors might include, for example, fostering economic and community empowerment and trust at the structural level, and promoting contact with people living with HIV and enhancing social support and adaptive coping at the individual level [15].

The present study takes a qualitative approach and focuses on two main research questions. First, within the context of a geographical urban HRA characterized by limited resources and economic inequality, we elicited and explored participants’ perspectives on how socio-demographic characteristics such as low socio-economic status, race/ethnicity, sexual orientation, sex/gender, and other such factors, all of which can produce stigma, intersect with HIV stigma to thereby shape the experience of HIV testing among this population at high-risk for HIV infection. Second, given that a substantial proportion of IR-HRA do, in fact, test for HIV, even if at sub-optimal rates, we sought to explore what factors promote HIV testing, and what types of health messages resonate most strongly with IR-HRA to promote HIV testing in the context of economic inequality and potential multiple, intersecting stigmas. A qualitative approach was selected to advance our understanding of the phenomena of interest from the perspectives of the population of interest; that is, an “insider” perspective, in order to produce findings with complexity and depth, rather than breadth [40, 41]. Further, the qualitative approach can produce unexpected or emergent findings, and thereby inform theory and future research.

## Methods

This paper draws on qualitative interview data with 31 adults with HIV-negative or unknown HIV status, diverse with respect to past and recent HIV testing experiences, who were participants in a larger intervention study that tested approaches to identifying heterosexuals with undiagnosed HIV infection in an HRA [11, 42–48]. The study received ethical approval from the New York University Langone School of Medicine Institutional Review Board.

### Brief description of the larger study

The larger study focused on heterosexuals considered at high-risk for HIV infection. Grounded in the National HIV Behavioral Surveillance (NHBS) studies, risk for HIV was conceptualized as largely a function of one’s connection to a high-risk geographical location rather than individual behavior [49]. This is because HIV cases are largely concentrated in high-poverty neighborhoods and past research shows that individual behavioral risk factors, including unprotected sex and substance use, do not fully explain racial disparities in HIV infection [49]. In the larger study we defined an urban geographical region with elevated rates of both poverty and prevalent HIV infection, called an HRA [48]. The HRA was located in central Brooklyn, NY, the borough in New York City, from among five boroughs, with the highest heterosexual HIV prevalence rate the time the study was planned [48]. We defined a core HRA comprised of seven ZIP codes with the highest rates of poverty and HIV prevalence, and a surrounding HRA comprised of 12 additional ZIP codes. Procedures used to define the HRA are described in more detail elsewhere [48]. Three HIV testing interventions were examined in the larger study, each conducted in the same HRA during the same time period (2012-2015). A study field site was established in the core HRA.

### Eligibility for the larger study and recruitment

African American/Black and Hispanic adults residing in the HRA were eligible for participation in the larger study if they were between the ages of 18 to 60 years of age; sexually active with at least one opposite sex partner within the previous year; and could conduct research activities in English or Spanish. Participants were recruited through respondent-driven sampling (RDS), a network-based method in which members of a target population are trained to recruit their peers for a research project [50]. RDS is a type of chain-referral sampling that limits the number of recruits, resulting in longer recruitment chains. These long chains increase the “reach” of the sample into hidden pockets of the population who might not otherwise present to a research study. In RDS, a small number of “initial seeds” start a recruitment chain by recruiting three to five of their peers, who then enter the study, and then recruit their own peers. Peer-to-peer recruitment continues until sample size goals are met.

The larger study was guided by the theory of triadic influence, a social cognitive theory emphasizing three streams of influence on heath behavior: individual, social- and structural-levels [51]. The interventions were culturally salient in that the structure of project activities (location, length) and content of sessions addressed the primary barriers to HIV testing experienced by IR-HRA (e.g., low perceived risk because of heterosexuality, low motivation to test, fear of HIV stigma). As noted above, there were three HIV testing interventions examined in the larger study. One intervention used RDS as its recruitment method and consisted of two intervention sessions, one to orient/engage participants and train them on peer recruitment, and the second providing confidential HIV counseling and rapid HIV testing using oral fluids. We refer to this as RDS-CTT (“Confidential Two-session Testing”). RDS-CTT was comparable to HIV testing services provided in a medical/clinical setting. Participants for the present study were drawn from RDS-CTT, the largest of the three interventions tested, and the other two interventions are described elsewhere [45]. A total of 3005 participants were enrolled into the test of RDS-CTT. Of these, 107 were initial seeds, selected to vary in age, sex, and race/ethnicity, who were directly recruited by study staff in 2012-2014 from public and street venues within the core HRA. These initial seeds began the peer recruitment chains. From this sample of 3005, 2351 participants were potentially eligible for the present study as described below because they declined HIV testing (*N* = 83) or tested negative for HIV (*N* = 2268). (Participants could decline HIV testing and remain in the larger study.) As part of the larger study, participants engaged in a structured baseline assessment lasting 60-90 min on socio-demographic and health history variables. Participants gave signed informed consent for all study activities including the qualitative interview and for audiotaping of interviews [48].

### Eligibility and sampling for the present study

In keeping with our interest on stigma and other barriers to and facilitators of regular HIV testing, participants were eligible for the present study if they were enrolled in the parent study and either declined HIV testing, or tested negative for HIV, during the larger study, as noted above. That is, participants with a past HIV diagnosis, or who tested positive for HIV during the study, were excluded from the present study. This design decision was intended to allow participants to keep HIV testing issues in the foreground during the qualitative interviews, themes that tend to be crowded out when issues of adaptation to an HIV diagnosis are primary. Participants were recruited for the present study using purposive sampling for maximum variation with respect to past HIV testing experiences, namely, ever having been tested at all in the past; engagement in regular, annual HIV testing; and participation in HIV testing during the larger study, with the expectation that those who declined HIV testing during the larger study would experience some of the greatest barriers to HIV testing generally. Our expectation was, given past research on barriers to HIV testing reported for this population, all participants, regardless of HIV testing experiences, would have insights into factors that promote or impede HIV testing in their communities. Interviews were conducted until saturation was reached on core constructs.

### Procedures for the present study

During 2012-2014, participants were contacted by phone after they had completed activities for the larger study and recruited for the qualitative interview. No participant declined to engage in the qualitative interview. Interviews were conducted in-person at a study field site located in the core HRA. Interviews followed a semi-structured interview guide that included main questions and probes, and interviewers were encouraged to attend to emergent topics. The study was grounded in an intersectionality perspective [52], and the theory of triadic influence [51], the multi-level social cognitive theory guiding the larger study. Grounded in these frameworks, the interview guide aimed to elicit perspectives on HIV testing behaviors including barriers and facilitators to regular testing at the individual-, social- and structural-levels of influence, and their interactions (e.g., low perceived risk because not in a primary risk category, medical distrust, competing priorities, substance use, fear of HIV stigma and other forms of stigma, structural barriers such as poor access to testing for HIV and other sexually transmitted infections, and facilitators of HIV testing). We focused retrospectively on participants’ past HIV testing experiences and their views on HIV testing more generally, and, since all participants were offered HIV testing in the larger study, and their thoughts and feelings about, and attitudes toward, this most recent HIV testing experience, whether they elected to be tested or not. Interviews were audio-recorded and transcribed verbatim. In order to protect participant confidentially, all names and personal identifiers were removed from the transcripts. Only pseudonyms are presented below, and some identifying details have been changed. Participants received $30 to compensate them for their time, plus funds for round-trip local transportation.

### Interviewing team and positionality

The qualitative interviewing team was made up of eight female masters and doctoral-level research study staff members from diverse racial/ethnic backgrounds (White, Black, Hispanic/Latina, and Asian), all trained in qualitative interviewing methods. Positionality challenges related to sex, gender, race/ethnicity, power, socioeconomic status, and privilege were intentionally addressed throughout the data collection process through regular peer and supervisor reflection and training, which focused on the manner in which these types of issues might impact the interviewing process and data collection [53].

### Data analysis

Qualitative data were analyzed using an approach that was both theory-driven and inductive using the Dedoose platform (Dedoose Version 7.0.23, 2016), taking a systematic content analysis approach [54]. The analysis process began with the generation and application of a robust set of reliable and valid codes. First, we created a “start code list” based on the research questions and domains of the theory of triadic influence model [55] and known primary barriers to HIV testing among IR-HRA (e.g., low perceived risk because heterosexuals are not in a primary risk category, medical mistrust, competing priorities, substance use, fear of HIV and other forms of stigma, social norms, and structural barriers such as access to testing for HIV and other sexually transmitted infections). We also attended to facilitators of HIV testing, strengths, and explored emergent codes. Since we expected stigma to be a latent, rather than explicit, code, we operationalized HIV-related stigma as negative beliefs, feelings, and attitudes towards HIV or people living with HIV [56]. These codes were comprised of labels or tags (containing one to several words) assigned to sections of text (words, sentences, paragraphs) that were accurately described by that code. First, a main data analyst read through four interviews and applied the start list codes to segments of text. This analyst created new codes based on emergent topics relevant to the main research questions or that were repeated in the transcript or across transcripts. Then, a second analyst independently coded a selection of excerpts already coded by the first coder. The two coders worked closely to discuss codes and establish inter-coder reliability by resolving discrepancies in coding by consensus. Through this grounded and inductive approach, additional codes emerged, and the codebook was further elaborated and refined [57]. Once consensus between the two analysts was reached on a consolidated list of codes and their definitions, both analysts re-visited the interview transcripts already coded and incorporated the final list of codes. The primary analyst then coded the remaining transcripts, the second analyst also coded approximately 25% of them, and discrepancies were resolved by consensus. Then, emphasis shifted from coding to identifying larger themes. The full analytic team comprised of the two data analysts and senior research staff formed an “interpretive community” [58], which engaged together in an iterative data analytic process. The analytic process was comprised of regular meetings to discuss the most frequent and resonant codes, relationships among codes, and their explicit and underlying, latent meanings, which were combined to form unifying themes. Codes and themes were deemed primary when they were introduced or discussed by numerous participants. Through this multi-step, collaborative data analytic process, HIV-related stigma and its intersection with other forms of stigma or socio-demographic characteristics, and the larger context of economic inequality, emerged as the most central and resonant themes, and the analytic process focused mainly on those themes. Thus, the approach taken was not strictly grounded theory but was considered inductive. This is because although the start code list and major research questions were guided by the theoretical models, we did not set out to test hypotheses, nor did we approach analyses with themes identified in advance. Methodological rigor of the analysis was maintained through an audit trail of process and analytic memos and periodic debriefing with the larger research team, which included experts in HIV testing issues, intersectionality, social/economic inequality, and stigma [59].

## Results

### Participants

As shown in Table 1, participants ranged in age from 22 to 60 years, with a mean age of 38 years (SD = 13.25 years). Most were male (74.2%), African American/Black (83.8%), and identified as heterosexual (90%). Two-thirds (66.7%) had a high school diploma or higher, but only 17.3% were employed part-time or full-time. Consistent with their residence in a HRA, indicators of low socio-economic status were common, including being unable to meet needs for basic necessities in past 12 months (73.3%). Half (48.3%) had been homeless in the past, and approximately half (46.7%) had been incarcerated in the past. Two-thirds had been tested for HIV in the past, prior to the larger study (66.7%), and these individuals had been tested nine times on average (SD = 11.63 times). Yet only 13.3% received regular, annual testing. Most (80.6%) accepted HIV testing during the course of their participation in the larger study.

**Table 1:** Socio-demographic and background characteristics (*N* = 31)

	Mean (SD) or %
Age in years (mean, SD)	38.65 (13.25)
Age range in years	22 – 60
Female sex	25.8
African American/Black	83.8
Hispanic/Latino	16.1
Sexual orientation	
Heterosexual	90.0
Bisexual	10.0
Education	
No HS diploma or GED	33.3
HS diploma or GED	53.3
Some college	13.4
Indicators of socio-economic status	
Receives gov’t benefits (e.g., SNAP benefits)	66.7
On Medicaid	86.4
Indication of extreme poverty (unable to meet needs for basic necessities in past 12 months)	73.3
Current employment	
Employed full-time or part-time	17.3
Unemployed or on disability	82.7
Ever homeless	48.3
Ever incarcerated	46.7
If ever incarcerated, past year incarceration	35.7
HIV testing history	
Ever received HIV testing prior to the larger study	66.7
(*If tested*) Times tested in the past (mean, SD)	9.0 (11.63)
Receives regular, annual HIV testing	13.3
Accepted HIV testing during the larger study	80.6

### Overview of results

Participants’ narratives revealed that HIV-related stigma, while manifesting at the individual level, was largely embedded in structural and institutional inequities, and also reified through cultural taboos regarding HIV in their communities. From an intersectional perspective, participants underscored the salience of low socioeconomic status and its potential connections to other forms of stigma, including HIV’s association with homosexuality and injection drug use, and potential of HIV stigma, therefore, to contaminate or threaten heterosexuality. While the effects of residing in a high-poverty area, and of low socio-economic status, on HIV testing behavior and stigma were apparent to participants, the importance of race/ethnicity as a factor was commonly implicit, and only occasionally made explicit. Further, despite the clear and marked desire among participants to avoid HIV stigma, participants identified a number of public health messages that served to motivate HIV testing, as well as characteristics of the HIV testing experience that increased their motivation to engage in regular HIV testing. Thus, we found while HIV stigma was indeed a potent barrier to HIV testing among IR-HRA, particularly because of its powerful interactions with other forms of stigma, most participants did commonly report they engaged in testing for HIV, and that they saw the utility of HIV testing. In fact, they identified a range of public health messages and characteristics of the HIV testing experience that fostered their own engagement in HIV testing, and that could potentially shape HIV testing experiences for others in their communities. Below we first describe participants’ perspectives on stigma-related factors that impede HIV testing in their communities, followed by the ways in which IR-HRA managed multiple stigmas and the reasons they engaged in HIV testing.

### Part I: Stigma and its intersection with other barriers to HIV testing

#### Stigma related to institutional marginalization

##### Stigma experienced from health care settings

Participants’ descriptions of HIV testing experiences reflected structural disadvantage they often faced in their larger communities. For instance, substandard conditions in HIV testing facilities, as well as an overall lack of services, were linked to the larger environment in the HRA, which was described as lacking in resources. Regarding the paucity of services typical in low-income communities, Joe, a 42-year-old African American/Black man, noted:

You don’t have the opportunity to go to places where they can get the AIDS test, especially in the poorer neighborhoods, you know what I mean? Because that’s the way it seems– it’s being taken away. You know, it’s just sad.

Moreover, existing HIV testing and health care services were described as run-down and inconvenient, with long waiting times for appointments. For example, participants who had previously tested for HIV described facilities as characterized by overcrowding, lack of privacy, an assembly-line approach, and dehumanizing interactions with health care providers who lacked compassion. In the following excerpt, Linda, a 36-year-old African American/Black woman, reported being treated like a nuisance by the staff at the city-run free clinic in her neighborhood, a geographical area she described as generally under-resourced.

Free clinic don't work. To me, you go in and you wait forever and it's mad dirty, and they rushing you... They want to see as many people as they can so they can go home. That's it. You positive, this is your drugs, take them. That's it. Get out.

Janel, a 40-year-old African American/Black woman, described a lack of confidentiality built into the physical spatial layout of the large bureaucratic health care facility in her neighborhood – a location that provides testing for HIV and other sexually transmitted infections.

When you’re coming in to be tested for a venereal disease, everything is in one area that you have to go in, so you always know who’s going to which person based on the person that came to get them from the back. So it’s kind of embarrassing because then you know that when you see the doctor come up front, he’s taking the people that are doing [HIV or STI testing]. When you see the nurse come up, she’s coming to take these people. Just to sit there and watch the people move in and out. It was like, wow. So there’s no sense of confidentiality. If you’re just sitting in the waiting room you basically can tell who’s doing what.

As a result of the physical layout of the clinic and attendant lack of confidentiality, Janel reported being embarrassed, and marked as someone who might have a stigmatized sexually transmitted infection including HIV, both from the health care providers and the other patients, simply by her presence in the clinic. Although these adverse experiences did not prevent Janel from accessing HIV in this case, they created an unpleasant experience for her, which, she suggested, could be avoided in a more carefully planned, less bureaucratic, or better-resourced facility. Yet, as participants were well aware, neighborhood poverty levels and the quality of health care facilities, at least their physical locations, were closely related. Nonetheless Janel did seek out and receive HIV testing despite her discomfort with the physical setting, underscoring her ability to tackle such obstacles to achieve a health-related goal. Nonetheless, Janel highlighted how a physical space can produce stigma and thereby impede health behavior.

##### A pervasive lack of educational resources and opportunities to understand HIV

Participants noted a lack of HIV-related education in the institutions in their communities, as well as a general unwillingness among their peers to openly discuss HIV-related issues such as prevention, testing, and treatment. This lack of education and open discussion, in turn, was seen as contributing to and perpetuating HIV stigma. Participants noted there was largely silence around HIV in their schools, where they had limited access to information about HIV risks, prevention, and testing services, or about critical advances in the treatment of HIV infection. This, in turn, contributed to the perception of HIV as a taboo subject in the community. For example, despite growing up during the peak of the AIDS crisis in an urban area, Peter, a 23-year-old African American/Black man, described receiving limited, if any, information about HIV in his high school sex education classes.

Basically, when I was in school, in health class, we basically talked about like other [sexually] transmitted diseases. We didn’t really never talk about HIV. We talked about syphilis and other diseases. If we did talk about HIV it wasn’t for a very long time.

According to Jeremiah, also a 23-year-old African American/Black man, the general lack of knowledge about HIV in urban communities such as his contributed to the fear of HIV, and the sense of HIV as a taboo subject.

The lack of education. If people don’t tell us and everything, or try to like, let's see, how do I put this? It’s like basically growing up in the ‘hood like, you fear what you don’t know. Like, oh, HIV, that sounds dangerous. I don't want to know about it. AIDS, that dangerous, I don’t want to know about it. So, all in all, nobody picks up that topic or sit down and take the time to explain it. And if they do sit down and take the time to explain it, it's like, I don't want to hear this, I’m leaving and they’ll storm out.

Thus from Jeremiah’s perspective, inadequate education on HIV within HRAs (“*the ‘hood*”) fueled fear of HIV, and contributed to individuals’ avoidance of engaging in discussions of HIV infection with members of their social networks, thereby impeding the acquisition of health-related knowledge. Thus, Jeremiah highlighted how fear of HIV, one critical aspect of HIV stigma, was generated and perpetuated by the very systems designed to educate and protect young people in the community.

In keeping with the silence around HIV in educational institutions, participants described HIV as a “*taboo*,” “*scary*” and “*touchy*” topic in their communities more broadly. Participants described the pervasiveness of fear, silence, and avoidance, and a lack of open communication around the topic of HIV, which, they noted, further exacerbated HIV stigma. When asked if he thought many of his friends had tested for HIV, Greg, a 22-year-old African American/Black man, connected their limited HIV testing experiences to an overall silence about HIV among his peers, and the fact HIV is not prioritized in his peer group.

I doubt they had a test… That’s not somethin’ we talk about. We don’t talk about, you know, have you ever been tested for HIV? I’ve known them for years too. And that… wasn’t even ever a topic. We don’t talk about, you know, have you ever been tested for HIV? Like I said, it don’t feel important.

Similarly, Peter, introduced above, described HIV as a “*scary*” topic that remained taboo and suppressed in the community.

I really don’t hear about HIV. And I feel it’s a scary topic to talk about. People don’t want to talk. People just want them and they doctor to know, you know? Don’t want everybody to know.

#### The complex intersection of multiple social stigmas

##### The association of HIV with stigmatized/marginalized groups

In part, present-day HIV stigma was linked to the early history of the epidemic, when HIV was predominantly understood as a disease that affected men who have sex with men and persons who inject drugs – two highly stigmatized social categories. As Damon, a 47-year-old Hispanic man, noted:

When the first HIV came out, you gotta remember it was the homo disease. You know, people used to discriminate [against people] like my own uncle. And then here he go, my [uncle’s brother], couple years down the line [he] gets it and he got it from shooting needles. You know, so here you go. The stereotyping and the discrimination. So I learned from young, you know, don’t discriminate about nothing.

Thus Damon was aware of past and present HIV-related discrimination, linked in part of HIV’s association with stigmatized social identities, but he also reported an evolution in his personal biases as he grew in age and experience.

A substantial number of participants noted that, particularly in the early days of the epidemic, HIV was referred to as “*the monster*,” or, in Spanish, “*el monstro*,” as Ramon, a 51-year-old Latino man, explained.

They used to call it the monster. They call it the monster too in Spanish – el Monstro. “*They got the Monstro; watch this person*.” So, you know, you hear things like that... People have a fear of fear itself.

Ramon went on to discuss how labeling and gossip about an individual’s HIV status perpetuated HIV-related stigma and exacerbated the fear of others within their communities. Ramon underscored how HIV-positive individuals not only experience the burden of the disease itself, but must also face the fear that others have of them as persons living with HIV. Moreover, Ramon related that he did not test regularly for HIV in the past because, as a heterosexual man, he felt immune to HIV and viewed it as a disease that primarily affected the gay community.

You know, you say, well, I’m heterosexual [so I do not need to test]. Because I’ve heard that too. You know, and I’m probably one that said it too. “I don’t got nothing to worry about.”

When asked about HIV in her community, Martha, a 46-year-old African American/Black woman, blamed the high local HIV prevalence rates on men who have sex with men, both condemning their actions and stigmatizing their identities.

So late at night you see them fags in the park. This is the HIV. This is where the AIDS come from. Because that park is affiliated with a number of faggots. Late at night—I got a window, I see it all. ‘Cause the park is right there. You got the faggots in the park. It’s a lot of gay in my community. Has a high risk over there.

Participants also distanced themselves from other groups they perceived as engaging in high-risk activities, such as persons who inject drugs and persons who engage in transactional sex. Like some other male participants, Michael, a 22-year-old African American/Black man, blamed the high rates of HIV in his community on young women, in particular those who engage in transactional sex.

Why do I feel like HIV is more in our neighborhoods, like, in the ghetto? Because of, like, the trends, fashion and stuff, like that in young girls and young guys. And young girls who want [fashion] are less fortunate and can’t get it. So they’ll do anything for it. They’ll probably do like almost anything for it, like as far as, like prostitution, and such, so forth and so on and so on, yeah.

Indeed, blaming the spread of HIV in their communities on other groups was common amongst participants: men frequently blamed women, and heterosexual men and women frequently blamed men who have sex with men, as well as persons who inject drugs and persons who engage in transactional sex.

##### Better not to know?

Due to the pervasiveness and potential high cost of HIV stigma for IR-HRA, participants commonly reported it was “*better not to know*” one’s HIV status, noting the fear of knowing one’s HIV status was a common impediment to regular, annual HIV testing. Avoidance of testing was most common among those with less up-to-date information about HIV infection and its treatments, as Jessica, a 26-year-old African American/Black woman described,

I felt like if you got it, it’s over. It’s finished. There’s no coming back from that. There’s no treatment. You could just go start digging your grave. Just lead to death. I’d rather not know. I don’t want to get tested and then find out that I do have it and then feel hopeless about my life.

Similarly, Samuel, a 55-year-old African American/Black man, reported testing for HIV fairly regularly, but suggested that many in his community avoid testing, particularly those with high rates of risk behavior, because they prefer not to know, due in large measure to fear of receiving a diagnosis of HIV.

Why they don’t want to get a test? Some people just might not want to know. You know? They might be afraid to know, knowing the lifestyle that they had, you know. Yeah. So they don’t want to know. They’d rather not know.

As articulated by Samuel, engagement in behaviors associated with the transmission of HIV can intensify the fear of testing HIV-positive, and avoiding HIV testing allows one to both push aside thoughts of those past risk behaviors, and quell fears about the possibility of having HIV.

We found avoiding HIV testing allowed participants to identify as seronegative and live their lives without the fear of being forced to grapple with HIV stigma. Michael, a 22-year-old African American/Black man, explained that by not testing for HIV, people can maintain the illusion that they are HIV-negative.

Some people find comfort in just not knowing. Just like when you don’t know, it’s just [that they] don’t know, so it’s like to them, they’re HIV negative [laugh]. If you don’t know then you’re HIV negative. When you don’t know, you’re just negative. You just say, you know what, I’m negative. So that’s the thing with not knowing. They’re gonna give their selves the benefit of the doubt.

Similarly, Jessica, a 27-year-old African American/Black woman who had her first HIV test during the larger research study, explained that she had not tested previously because she felt safer not knowing her status, expressing a sense of fatalism.

I’d rather not know. I don’t want to know it. If that’s the case just let it sneak up on me. But I ain’t want to know. I don’t want to get tested and then find out that I do have it and then feel hopeless about my life. I mean I want to continue living out my life and then whatever happens, happens.

Yet despite the desire not to know her status, Jessica elected to be tested for HIV during the larger study, highlighting how fear of HIV does not preclude engaging in a dreaded health behavior.

On the other hand, avoiding HIV testing and tamping down fear was not without cost, as reflected in the marked experiences of relief reported by participants after testing negative for HIV. Damon, described above, put it this way:

Well, I was relieved, you know, ‘cause I did take the test. And we sat down and it was negative. I felt 100 pounds lighter. You know, it was relief. So I felt good. You know, it was a blessing to know that in that state I’m good and that everything was explained and that it was painless and it was simple to understand. So it was a big relief to know, especially when it came back negative.

Although participants universally experienced anxiety and trepidation when being tested for HIV, these types of experiences were most pronounced for those who tested for HIV rarely or not at all in the past, and who reported carrying the heavy burden of fear of possible HIV infection over long periods of time.

##### Fear of loss of social relationships is a major impediment to testing

The potential loss of social relationships, including romantic and sexual relationships, was a common fear associated with HIV testing and learning one was infected with HIV. Like many other participants, Michael related that disclosing an HIV-positive status—and experiencing the associated stigma—would be just as, if not more frightening, than having the disease. When asked if, hypothetically, having HIV or having people know he had HIV was scarier, Michael replied:

This is probably gonna sound weird, but havin’ people know it, to me that’s scarier than havin’ it. ‘Cause havin’ it [without telling others], I mean I can still—they would hang around with me and stuff, and I can deal with it like how I wanna deal with it, you know.

Thus, Michael articulated one of the main reasons that HIV stigma was so feared and HIV was avoided: HIV infection could mean the loss of vital social relationships, and result in social isolation. For Michael, and many other participants in this study, the possibility of social isolation and loss associated with an HIV infection was insupportable, and therefore, HIV testing was viewed with trepidation. In other words, HIV stigma could result in serious, real world negative effects on vital social relationships.

Similarly, Isaiah, a 45-year-old African American/Black man who declined HIV testing during the study, preferred “*not to know*.” He explains his dilemma as follows:

I'm really up in the air with [getting tested for HIV]. But, at the same time I really do want to know, you know, and at the same time I don’t want to know. So it's like a 50/50 thing. You know, I can’t go home to my better half—how can I lay with you? Like, you know, how could I lay with you knowing and we have unprotected sex? You understand? So, in other words, if we’re having unprotected sex, you have to be—‘cause bodily fluids, you know, and that’s how it’s transferred from what I understand.

Indeed, in the present study men in particular reported being influenced by the fear that HIV infection could interfere with critical sexual and romantic relationships – a loss that for many seemed intolerable.

#### Stigma in the context of low socioeconomic status

##### HIV stigma as exacerbating hardships related to poverty

Participants identified several other common reasons why the possibility of HIV infection, and HIV stigma, were feared, or even considered impossible to manage. Jeremiah and Linda, both introduced above, explained that since people in their community face extreme difficulties like homelessness and gun violence, they just cannot take on the extra burden of a seropositive HIV status. As Jeremiah noted,

It's like, if you below poor, then the average poor person considers it, like, why would I want to go get HIV tested to make my life even worse than it already is? Like, I'm already living on the streets. I'm out here freezing my butt off every winter and now on top of that, I got HIV. That's crazy. Now I can't put it up with it. So, they just don't. They rather not know and live like that, than knowing and—and probably live with it.

Linda described the dilemmas that IR-HRA experience as follows:

Yes, because with all the information, like, you can get condoms from anywhere so, like, to me, you would think [local HIV rates] went down, not up. With so many people dying, we always got to worry about getting shot, now I got to worry about you have AIDS too?

Thus for IR-HRA with limited economic means, in communities with limited resources, and serious, potentially life-threatening concerns related to survival, HIV stigma is a burden many feel they cannot shoulder, and HIV testing, therefore, is not a priority. Additionally, several participants indicated they did not get tested for HIV regularly because testing HIV-positive would derail their hard-fought achievements. Isaiah, introduced above, declined HIV testing during the study, and described his ambivalence about knowing his status in the following quote:

Basically, alright, I'm gonna be honest with you. I don't want to take the test because [having HIV] would really detour my life right now. It would really like throw me off course. I'm in the process of doing the right thing, you know? You know, when you're young, you do bad things. You get older, you start realizing things, but at the same time it's a part of me that's saying, why not [test]? If you feel so confident, you know, why not take the test and see? I'm really up in the air with it. But, at the same time I really do want to know, you know, and at the same time I don't want to know.

Thus Isaiah uncovered another reason why HIV testing might be avoided by IR-HRA; namely, it was a risk that some are unwilling to take, particularly as they age and begin work to improve their lives, related to the belief that HIV would “*detour*” one from his or her path, for some of the reasons noted above, linked to HIV stigma.

### Part II: Managing and overcoming HIV stigma

Above we described participants’ perspectives on the serious and complex reasons why they, and their peers in their communities, typically fear and avoid HIV testing. Yet many IR-HRA do in fact test for HIV, some once, some sporadically, and some regularly. In this section, we uncovered and described participants’ perspectives on how they managed fear of HIV stigma, and what types of public health messages best promote HIV testing in this population.

Above we noted that participants highlighted a prevalent attitude in their communities that it is “*better not to know*” one’s HIV status, to prevent taking on HIV stigma with might “*detour*” lives or destroy friendships and sexual/romantic relationships. However, we found it was just as common for participants to note that it is, in fact, “*better to know*” one’s status. As Peter, described above, put it,

All my friends are saying they don’t want to, they just don’t want to know [their HIV status]. But it’s better to know than not to.

Further, Jessica, described above, noted the importance of detection of HIV, because of the advantage of early treatment for HIV infection.

[I used to think] it’s better not to know. Now I’m feeling like it’s better to know, ‘cause you don’t want it to move along and then you catch it in a late stage where you can’t do nothing about it. It’s better to know as early as possible so you can take care of it and still live your life. If the disease progresses, you know, then it start breaking out in your immune system. It gets to that point it’s not HIV no more. It’s AIDS and it’s a totally different monster.

Overall participants fell into one of two camps with respect to knowing one’s HIV status, namely knowing versus not knowing. Further, some participants represented both viewpoints in the same interview, highlighting the complexity of barriers to HIV testing. For others, such as Jessica, their perspectives on HIV testing evolved over time. We found, in part, participants’ viewpoints on whether there were advantages to knowing their HIV status were related to factors such as their current life circumstances and whether they believed they would be able to manage an HIV diagnosis at this time, perceptions of past risk behavior (and whether they were likely infected with HIV, or could be infected, such as if they were “*promiscuous*”), and their relationship status. With respect to the latter factor, we found being in a romantic and sexual relationship could promote HIV testing in some cases, and impede it in others. For example, Isaiah, introduced above, elected not to test during the larger study, in part because of how finding out he was living with HIV might affect his partner.

I have one partner that we've been together five years now and I don't want her to think because I'm getting tested that there's something wrong with me or herself. Because you know, women are—you know, they tend to think about there's something wrong with me for you to go do that unless you cheating on me or, you know. It gets real dicey.

Thus, for Isaiah, receiving HIV testing could signal to his partner that he has been unfaithful – an accusation he wished to avoid. In other cases, participants who were not currently in relationships declined HIV testing until romantic partnerships were solidly in place, some decided to obtain HIV testing together with romantic partners, and others experienced their romantic partnership as a reason to test for HIV.

### HIV testing as an altruistic act

Participants commonly noted they elected to be tested for HIV as a means of helping the community, by potentially preventing forward transmission of HIV if they were found to be infected. When asked why he chose to be tested for HIV as part of the larger study, Josue, a 31-year-old Hispanic man, noted

Cause I wanted to know. I’m not selfish. I didn’t wanna get somebody infected. I’m not going to be selfish and meet somebody and she’s healthy and be selfish and just pass something without knowing because I don’t – I’m scared to check it out. But, what changed my mind again was knowing I’d played the Russian roulette. Knowing that I don’t wanna end up being selfish again in passing it to an innocent person maybe who got me. You know, caring about other people. Not just myself or being just selfish about myself. ‘Cause I won’t sit here and tell you I don’t care. I do. And I care about tomorrow now. I care about the next person and the next girl, you know.

Moreover, learning that African American/Black and Hispanic heterosexuals in HRAs are at higher risk for HIV than their peers who do not reside in HRAs can motivate testing, in part by raising community awareness, and also challenging the notion that heterosexuals are not at risk for HIV. As Peter, described above, highlighted,

The HIV is around in this community and you know, poorer communities. So that’s what made me like really wanted to get tested. Yeah.

### High-quality HIV testing experiences

In the context of fear of multiple and often intersecting stigmas, aspects of the HIV testing experience could either promote or impede uptake of this health behavior. Participants noted that understanding that HIV testing is free, confidential, and can be conducted with an oral swab, rather than a blood draw, increased interest in testing. Participants also highlighted the importance of a high-quality “*professional*” HIV testing experience, where time was taken to explain the procedure, as Isaiah described:

Look a person in the eyes. Don't turn your head away, you know, make them feel like you actually concerned about them.

Moreover, emphasizing the voluntary nature of HIV testing was seen as a desirable feature of the procedure, and increasing comfort through support for one’s choices, “*professionalism*,” and “*respect*,” as Damon noted.

[It’s good if] you’re not going to be looked down if you don’t do this and if you don’t feel like answering, so [they should] make you feel as comfortable. So [the testing experience should be] very professional, very respectful, and you can tell that [they] care.

### Allaying concerns about living with HIV infection

As participants noted above, HIV is not commonly discussed in their communities, and HIV education was generally poor. Yet HIV treatment has evolved dramatically since the early days of the epidemic, and HIV antiretroviral regimens are increasingly tolerable, with minimal side effects, and also highly effective. Many participants had family members who died from the consequences of HIV infection, including in the early days of the epidemic. In fact, participants did commonly note progress in the treatment of HIV, particularly as more of their peers and community members disclosed living with HIV, and this awareness generally increased motivation to test for HIV. Tonya was a 59-year-old African American/Black woman who described it as follows:

I have so much love for Magic Johnson. He remembers the time when he was taken out-I don’t know, dozens, there was dozens of pills that he had to take back in the day, but now it’s down to three. You know, it’s about progress. We’ve come a long way, you know.

Coming into contact with HIV-infected persons who were open about their HIV status and willing to discuss living with HIV was seen as a potent intervention against stigma by many, as Joe, a 42-year-old African American/Black man described after seeing such a presentation, contrasting present-day openness about HIV with past shame and fear.

People [living with HIV sharing their stories] – they were so confident about themselves, you know? And they shared so easily about their disease, and not being ashamed of it. Because back in the day, you were ashamed of that stuff. You didn’t tell anybody you had it, you know? For fear of, you know, “oh, my God, stay away from him.”

The fact that HIV was no longer a “*death sentence*” was a common theme in this analysis, including the fact “*that you can live with it, and you can live a normal life*.” Further, there was growing awareness that HIV-infected persons with undetectable HIV viral load levels cannot transmit HIV to their sexual partners, that is, the notion that **“***if you take your medications, it’s hard for you to pass it on to your partner.*” Thus, participants noted, as a consequence, persons diagnosed with HIV did not necessarily have to lose vital romantic and sexual relationships. Participants highlighted a number of public health messages that served to increase their comfort with HIV testing and motivation to test. As Joe, introduced above, stated:

You can live with [HIV], and you can live a normal life. You know? As long as you take care of yourself. You take your meds, and take care of yourself, you know? I mean, you can live a normal life. And you can actually – you can still have a sex life, and everything. I mean, you really could. Of course, it may be a little harder for you than a normal person. But, I mean, if you tell your partner, and you’re protected, and you’re protecting, you know – and she’s understanding.

In the third decade of the HIV epidemic, participants described the potency of HIV stigma as a barrier to HIV testing. But at the same time they identified a number of public health messages, interventions, and experiences that served to reduce stigma and increase motivation for HIV testing, highlighting the dynamic nature of both the HIV epidemic and HIV stigma.

## Discussion

Using an intersectional framework focused on structural stigma, the present study uncovered and explored how factors that promote and impede regular HIV testing operate in an understudied population at high-risk for HIV: African American/Black and Hispanic heterosexual individuals residing in an urban HRA, a geographical location where institutional and personal resources are limited. This framework allowed us to explore ways in which multiple and overlapping structural stigmas experienced by many IR-HRA directly affect individuals’ decision-making with regard to HIV testing [26, 60, 61]. As Crenshaw [52, 62] discusses in her work on violence against women of color, by remaining attentive to how multiple social identities inform and reinforce one another, an intersectional approach allows for a nuanced and powerful understanding of how individuals are uniquely located at the nexus of a variety of often marginalized social positions. For participants in this study, race/ethnicity, class, sex, and sexual orientation all intersect to inform individuals’ attitudes toward HIV in general, and profoundly shape the ways they think about, experience, grapple with, and then, either avoid or engage in HIV testing.

### The compounded effects of HIV and other structural stigmas

Consistent with previous research [30, 63, 64], we found community-level poverty creates conditions whereby HIV-related stigma acts as a deterrent to HIV testing among IR-HRA, as a result of their interactions with substandard health care and educational institutions, often resulting in a fear of and silence about HIV. Participants’ HIV testing experiences are commonly negatively characterized by overcrowding, bureaucracy, a lack of privacy and confidentiality, and a lack of empathy. For many IR-HRA, the healthcare system perpetuates a ‘continuum of harm’ [65] wherein interactions with health care providers and HIV-testing sites maintain and reproduce institutionalized systems of race- and class-based disparities in health and health care [29]. Negative HIV testing experiences in these communities confirm and reify perceptions of HIV as a stigmatized condition, and participants often feel vulnerable to stigma within the very institutions whose mission it is to provide respectful and high-quality medical care and services. Moreover, participants highlight how silence around HIV-related issues in secondary schools cement the perception of HIV as a taboo topic. Lack of HIV-related education also leads to confusion and fear regarding which social groups are at risk for HIV, as well as a lack of awareness of recent advancements in HIV treatment. Regarding the former, many participants still most closely associate HIV with homosexuality, intravenous drug use, and transactional sex, all of which are described in a heavily stigmatized manner. Regarding the latter, many participants continue to regard HIV as a death sentence, and associate being HIV-positive with a host of socially undesirable physical symptoms. These experiences and perceptions serve as impediments to IR-HRA receiving regular HIV testing services.

There is a substantial literature underscoring the importance of understanding how structural stigma operates at both the macro- and micro- levels [27, 28, 61, 66]. For participants in this study, the complex intersection of racial/ethnic minority status and low socioeconomic status, including disproportionately high levels of incarceration and periods homelessness experienced in the past by many, prove to be a highly challenging source of conflict and stress. As a result, some IR-HRA actively avoid HIV testing, stating that it is “*better not to know*” one’s HIV status, since receiving a diagnosis of HIV would create additional sources stigma and potential discrimination, a loss of already limited resources, and a diminished status within their communities. Indeed, participants commonly view HIV-related stigma as a comparable, and at times a more dangerous threat, than the burden of being HIV-infected, leading individuals to actively avoid regular HIV testing. Participants describe that by further potentially exposing them to social and structural stigma, discrimination, and exclusion, they fear HIV-related stigma will intolerably compound the hopelessness and vulnerability faced by those located at the intersection of multiple marginalized identities. For IR-HRA, a potential HIV diagnosis carries with it not only a lifelong and potentially terminal disease, but also the possible loss of social connections, social and tangible resources, and social status. Even more troubling to participants involves the ways in which an HIV-positive status would hinder their hard fought personal achievements and thwart their sense of a more optimistic future. These potentially devastating losses are the filters through which many IR-HRA interpret the health care choices they make.

### The association of HIV and other marginalized social categories

Further, participants commonly seek to create and maintain distance from associations between HIV and homosexuality, intravenous drug use, and transactional sex. For IR-HRA in this study, one of the only non-marginalized social categories within which they are located is that of a heterosexual sexual orientation. Heterosexuality consequently serves as an important stabilizing identity that is threatened by its possible intersection with HIV seropositivity. Similar to past studies, we found even among those who are unaware of their HIV status because they avoid HIV testing, participants often further seek to avoid HIV stigma by attributing the spread of HIV primarily to other marginalized groups such as persons who inject drugs, those who engage in transactional sex work, or gay men [27, 67]. Because for many IR-HRA HIV-seropositivity carries with it these heavily stigmatized identificatory associations to homosexuality, transactional sex, and intravenous drug use, its intersection with heterosexuality is seen as potentially disruptive and threatening, leading to avoidance. This is especially relevant given the already fraught intersection with race/ethnicity and class, all of which have serious implications for IR-HRA’s interest in testing for HIV.

### Resiliency and managing stigma

As previous research has demonstrated, however, individuals with multiple, overlapping marginalized identities nevertheless develop strategies for managing stigmatization [15, 68, 69]. Indeed, we found that despite anticipating additional stigmatization, discrimination, and marginalization as a result of even visiting an HIV testing site, IR-HRA nonetheless commonly endorse testing for HIV is an important health behavior. Further, most in the present study received HIV testing in the past, although not typically on the recommended annual schedule. For some, these strategies to overcome the fear of stigma include a recognition of the advantages of early treatment for HIV, a desire to avoid progression from HIV to AIDS, and an altruistic desire to prevent forward transmission of HIV to others should they be found HIV infected. Others imagine themselves as resilient in the face of a possible HIV diagnosis, and speak positively about their abilities to draw on the stability provided by strong romantic and other social relationships. Moreover, tapping into knowledge provided by peers as well as public health messages (such as those incorporated into the larger study from which participants in the present study were recruited), including regarding the ability to maintain a positive, healthy life and to continue to build meaningful relationships, many IR-HRA express a desire to test for HIV regardless of the many obstacles they face. Indeed, past research suggests public health strategies to reduce HIV stigma at the community level, and to improve the quality of the HIV testing experience, including with respect to the physical locations where HIV testing is provided [70] could have a potent effect on increasing HIV testing in at-risk populations. Reducing stigma and improving the quality of the HIV testing experience would thereby reduce the need among individual IR-HRA to manage complex, distressing, and conflicting thoughts, fears, and worries in order to receive a needed health service [71].

### Advantages and disadvantages of the balanced data analytic approach

We analyzed data using a systematic content analysis approach that balanced theory-driven and inductive perspectives, guided by intersectionality theory and the theory of triadic influence. Indeed, a truly grounded theory approach is not generally feasible in cases such as this when a qualitative study component is embedded in a larger study, because the larger study will naturally be rooted in theory and strive to answer over-arching research questions. Indeed, we believe the theoretical models’ focus on multiple levels of influence on health behavior (namely, individual-, social, and structural-levels) and intersectionality played an important role in advancing our knowledge on the complex and inter-connected ways stigma operates in this population. Thus the advantage of this balanced approach, we believe, is that it facilitated development of themes on this complex topic across multiple levels of influence for this intersectional population. One possible disadvantage, however, is that the approach, which begins with a start code list, could have caused us to miss important codes, and therefore, critical themes as well.

### Limitations

The present study has several limitations. One general limitation is the purposive sampling method, which may limit its generalizability to the population of IR-HRA as a whole. Yet purposive sampling is consistent with the goals of qualitative research, which aims for depth rather than breadth. A second limitation has to do with the peer-to-peer recruitment method used in the larger study, where recruiters informed their recruits about the goals of the larger study, including the HIV testing component, before the recruit presented to the study. Thus, the sampling frame used for the larger study may have biased the sample towards those with more favorable attitudes toward HIV testing, since those with a very strong aversion to testing may not have enrolled in the larger study. Further, although not all participants in the present study had been tested for HIV in the past, the fact that those enrolled in the larger study received culturally salient HIV counseling and were offered HIV testing may have served to influence their attitudes toward HIV testing, perhaps in a more favorable direction in some cases. Yet despite these sampling issues, participants included in the present study, sampled for maximum variation, evidenced heterogeneity in HIV testing patterns, and uncovered and unpacked a wide range of perspectives on HIV testing, in depth, suggesting that socially desirable or otherwise biased responding were minimal. Moreover, the relatively small sample size did not allow us to examine gender differences in detail, a gap that future studies on this topic can address. Last, the present study did not include respondent triangulation, such as interviews with health care providers or other stakeholders. Indeed, such triangulation would have allowed us to examine HIV stigma from different perspectives and thereby validate results through cross verification [72].

### Implications for increasing regular HIV testing among IR-HRA

Study findings have implications for programs, policies, and enhancements to HIV testing services to reduce stigma in HRAs and thereby foster regular HIV testing among IR-HRA, as we review in Table 2. At a macro level of influence, poverty is a critical driver of HIV stigma. As Link and Phelan (2001) have noted, stigmatizing processes affect multiple domains of people’s lives, and can have dramatic adverse effects on critical domains such as earnings, housing, criminal involvement, health, and life itself [26]. The present study suggests that populations such as IR-HRA with limited economic resources and options, along with connections or potential connections to other stigmatized categories, have greater motivation and need to avoid acquiring other stigmatized categories compared to populations with more resources. Further, Tsai and colleagues (2013) highlight that living with HIV infection has adverse health and economic impacts, and undermines HIV-infected persons’ abilities to maintain their full economic contributions to family and community life and engage in reciprocal exchange [73]. Yet societal-level policies can reduce poverty [74], and social/behavioral interventions, such as livelihood or conditional cash transfer programs can reduce the stigma of HIV, or fear of stigma of HIV, by directly targeting poverty, to increase individuals’ life chances [73, 75].

**Table 2:** Practical recommendations that emerged from the present study

*Community-level stigma reduction approaches* Conduct multi-component community-level interventions in HRAs to reduce stigma *Changes needed to organizations and systems IR-HRA encounter* Provide high-quality HIV education in schools and other setting IR-HRA may encounter Design the physical layout, and look and feel, of health care settings to minimize stigma *Locating the population for HIV testing* Conduct peer-to-peer active outreach approaches in HRAs to engage IR-HRA in testing *Aspects of the HIV testing experience* Provide reassurance that confidentiality will be protected Provide compensation for HIV testing Provide counseling/testing approaches tailored to heterosexuals at high-risk for HIV As part of testing, highlight the provision of linkages to HIV care, and the availability of support during the process of adapting to a new diagnosis *Specific health messages to combat stigma and motivate testing* Highlight the community-enhancing nature of HIV testing, to harness altruism Provide health education on HIV, its psychosocial consequences, and treatments Provide exposure to culturally similar peers living with HIV who are thriving Incorporate messages into HIV counseling that fill gaps in knowledge and address fears, including: HIV testing is free, voluntary, and confidential HIV is not a death sentence HIV medications are available, effective, and highly tolerable One can live a long and healthy life with HIV HIV does not mean the end of sexual and romantic relationships	

Frye and colleagues (2017) implemented a multicomponent intervention via workshops, space-based events, and bus shelter ads delivered to community-based organizations and neighborhood residents in a high HIV prevalence, primarily African American, Black and/or Afro-Caribbean, neighborhood in New York City, taking an intersectional perspective. The study did not find a significant treatment effect on HIV stigma and homophobia among residents of the neighborhood where the intervention was implemented compared with community controls in a separate neighborhood. However, HIV testing increased by 350% at the testing site located in the intervention community [76]. Similar to the present study, the Frye study highlights the potency of stigma as a barrier to HIV testing, but also that residents in these geographical locations manage stigma to engage in health behavior. Thus experiences of stigma may not have changed in response to the intervention, but findings suggest community residents managed stigma more effectively [76]. Yet the authors note that evaluating community-level interventions is challenging [76]. Nonetheless, community-level stigma reduction interventions may hold promise for IR-HRAs.

The present study yielded recommendations for the institutions in HRAs, such as schools, community-based organizations, and health care facilities. Since the lack of high-quality HIV education in HRAs fosters HIV stigma, such organizations are well-positioned to play a leadership role in changing the climate of fear and trepidation surrounding HIV testing. Design the physical layout, and look and feel, of health care settings to minimize stigma. Yet while other studies have noted the importance of the physical layout and its ability to create or reinforce stigma with other populations [77, 78], efforts to modify such settings to reduce stigma are scant. Intervention approaches such as Community-Based Participatory Research [79] hold promise in the effort to address public health problems such as these. Further, study findings suggest a number of ways the HIV testing experience itself can be improved, along with specific health messages that can be incorporated into the HIV testing experience to combat stigma and motivate testing, as we note in Table 2. In keeping with the complex and pervasive nature of HIV stigma, simultaneous interventions at multiple levels of influence may be needed to reduce HIV stigma in HRAs.

## Conclusions

HIV stigma has been recognized as a major barrier to HIV-related health behaviors since the earliest days of the epidemic. However, compared to other risk groups such as men who have sex with men, less is known about the effects of HIV stigma among heterosexuals at high-risk for HIV, the majority of whom are African American/Black and Hispanic. The present study addresses this gap and underscores the enduring potency of HIV stigma in this population, including because HIV stigma interacts with other stigmatized identities. HIV stigma is generated and reinforced by structures in HRAs, such as school and health care systems, and its influence stems in large part from the fear that HIV infection will destroy vital relationships and reduce life chances. Indeed, HIV stigma may be a greater threat to populations with the fewest buffering resources compared to their well-resourced peers, highlighting how HIV stigma functions differently at varying levels of socio-economic status. Yet IR-HRA commonly overcome stigma and engage in HIV testing. Participants identified a number of public health messages and strategies that can be harnessed to encourage HIV testing in HRAs. Thus, despite the success of many IR-HRA in overcoming stigma and engaging in HIV testing, stigma-reducing policies and interventions at the levels of communities, institutions, social networks, and individuals have potential to reduce the burden of stigma among IR-HRAs.

## Abbreviations

ART: Antiretroviral therapy
HRAs: High-risk areas
IR-HR: Individuals residing in HRAs
RDS: Respondent-driven sampling
RDS-CTT: Respondent-driven sampling with confidential two-session testing
